# Association of Fatality Risk With Value-Based Drug Pricing of Epinephrine Autoinjectors for Children With Peanut Allergy: A Cost-effectiveness Analysis

**DOI:** 10.1001/jamanetworkopen.2018.4728

**Published:** 2018-11-16

**Authors:** Marcus Shaker, Matthew Greenhawt

**Affiliations:** 1 Section of Allergy and Immunology, Dartmouth-Hitchcock Medical Center, Lebanon, New Hampshire; 2 Dartmouth Geisel School of Medicine, Hanover, New Hampshire; 3 Children’s Hospital Colorado, Section of Allergy and Immunology, University of Colorado School of Medicine, Aurora

## Abstract

**Importance:**

The high cost of self-injectable epinephrine autoinjectors may represent a barrier to community anaphylaxis management. Value-based pricing can provide a benchmark for rational epinephrine autoinjector costs.

**Objective:**

To define value-based pricing of community epinephrine autoinjectors.

**Design, Setting, and Participants:**

In an economic evaluation study using a cost-effectiveness birth cohort model over an extended 80-year time horizon, Markov simulations of children with peanut allergy evaluated cost ceilings for value-based epinephrine prices in peanut allergy. Simulation inputs included all-cause age-adjusted mortality (2013 US life tables), 2013 published food allergy fatality rates, 2017 rates of autoinjector device carriage, and 2016 published market costs of self-injectable epinephrine. All costs were expressed in 2018 US dollars and discounted at 3% per annum.

**Exposures:**

Cohorts of children with peanut allergy prescribed epinephrine autoinjectors were compared with those not receiving personal epinephrine prescriptions. Children without epinephrine autoinjectors assumed 10-fold to 100-fold fatality risk increases. Costs were evaluated from a societal perspective.

**Main Outcomes and Measures:**

Fatality risk, quality-adjusted life-years, and incremental cost-effectiveness ratio.

**Results:**

A total of 100 000 simulated infants with peanut allergy entered each strategy, with two-thirds of the group receiving annual personal epinephrine prescriptions and using those devices appropriately when indicated. Over the time horizon, the cost of anaphylaxis preparedness and treatment in those with personal epinephrine devices was $25 478 (95% CI, $25 399-$25 557) compared with $654 (95% CI, $645-$663) for those without personal epinephrine, resulting in an average food allergy fatality of 0.00056 (95% CI, 0.000414-0.000706) per patient prescribed self-injectable epinephrine and 0.00148 (95% CI, 0.001242-0.001718) in those not prescribed self-injectable epinephrine. The value-based price (incremental cost-effectiveness ratio, $100 000 per quality-adjusted life-year) for personal epinephrine based on 10-fold fatality risk difference was $24. At a market cost of $715 per twin pack, the autoinjector incremental cost-effectiveness ratio was $2 742 697 per quality-adjusted life-year. If a hypothetical fatality risk protection from personal epinephrine was modeled at 100-fold, the value-based price ceiling for a personal autoinjector was $264.

**Conclusions and Relevance:**

In a simulation of children with peanut allergy, a value-based epinephrine cost has a ceiling of $24 for a personal autoinjector, even at an exaggerated fatality risk.

## Key Points

**Question:**

Given that wide variation exists in costs of epinephrine autoinjectors used to treat community anaphylaxis, can we define a value-based price for such devices?

**Findings:**

In this economic evaluation of community anaphylaxis treatment, the ceiling value-based price for an epinephrine autoinjector was $24, assuming a personal autoinjector prescription decreases the risk for food allergy fatality by 10-fold.

**Meaning:**

Epinephrine is central to managing anaphylaxis, and $24 personal autoinjectors are cost-effective for community-based anaphylaxis management.

## Teaser

This economic evaluation uses a cost-effectiveness model with inputs including allergy fatality rates, device carriage rates, and market device costs to investigate the value-based price of an epinephrine autoinjector for children with peanut allergy.

## Introduction

For children and adults who live with food allergies, personal self-injectable epinephrine devices are an important part of anaphylaxis preparedness. While accurate diagnosis of food allergy allows appropriate avoidance, unintended allergen ingestions still occur and are a source of concern. Epinephrine availability is crucial for individuals with an identified potential life-threatening allergy or condition that predisposes them to anaphylaxis, and it is recommended that at-risk individuals maintain an epinephrine autoinjector device on their person or in close constant proximity.^1,2^ While early administration of epinephrine during anaphylaxis is facilitated by undesignated epinephrine in some schools and public places and on ambulances, maintaining an personal autoinjector provides more immediate access to this important medication. At present, 49 of 50 states have laws that either permit or mandate schools to stock undesignated units in the event that someone experiences anaphylaxis at school and either is not aware of his or her condition or does not have access to his or her own device.^3^ This is important because even some individuals with identified food allergies neglect to keep epinephrine available at all times.^4^ Although preventable, food allergy fatalities are fortunately rare. A 2013 systematic review and meta-analysis suggested that annual food allergy fatality in at-risk individuals is between 1.81 to 3.25 persons per million.^5^

While the exact risk reduction associated with carrying personal epinephrine devices is unknown (to our knowledge, such a study has not been conducted and may not be considered ethical to conduct), it is widely accepted that this practice is a bedrock of food allergy management because it promotes early access to emergency treatment for severe reactions. The epinephrine autoinjector is an old device, dating back to modernization of atropine injection devices for battlefield use in the 1970s. These advances were developed and financed by the US government and were long ago paid off.^6^ The predominant device design is a spring-loaded, pressure-activated injection, and such devices are estimated to have a production cost of a few dollars, not accounting for licensing royalties.^6,7,8,9^ The AUVI-Q, the newest branded device on the market, is a notable exception to this, as it contains a lithium battery, retracting needle, and a voice-activated mechanism, differentiating it from the standard spring-loaded autoinjector sold as EpiPen and the generic form of EpiPen, the Adrenaclick.^10^ The design for EpiPen (and its generic) was revamped in 2011 to a more ergodynamic mold and involved a sheath that covers the needle.^11,12^ The retail purchase price of autoinjectors is heterogeneous and often expensive even with insurance, in some instances reaching $690 per dual-pack package (2016 dollars), although retail device costs rarely exceeded $100 before 2010.^9,12,13^ Devices expire within 18 months and are generally renewed annually, although in August 2018 the US Food and Drug Administration (FDA) temporarily extended the expiration an additional 6 months for specific product batches given device shortages.^14^ It was only recently that programs were developed to assist widespread distribution of epinephrine autoinjectors in select populations (eg, schools that have legislation allowing for undesignated stock units to be maintained) without cost (Epi4Schools, coupon programs).^15,16^ The cost of the epinephrine contained in the autoinjector is very cheap. When supplied in ampule vial form, epinephrine costs approximately $1.^17^

The issue of value-based drug pricing has been recently reviewed.^18^ Value-based pricing is a method of drug pricing in which the drug cost is based on the magnitude of benefit it provides to those who use it, and perhaps to society as a whole.^18^ Because value-based pricing of personal epinephrine autoinjectors has not been performed, this study was undertaken to further characterize optimal epinephrine autoinjector pricing from this perspective, which may help to inform policy makers given recent steep price increases for this product.

## Methods

We compared simulated birth cohorts of children with peanut allergy, with and without personal epinephrine autoinjector prescriptions (n = 100 000 per group), using computer-based mathematical microsimulations (TreeAge Pro) over an 80-year time horizon (eFigure in the Supplement). Markov models facilitated incorporation of annual costs and ongoing risks of peanut exposures and reactions, as well as incremental risks of fatal food allergic reactions. Markov models are an optimal tool to compare patients who experience recurrent probabilistic risk by allowing simulation of the natural history of conditions characterized by different health states.^19^ Base-case assumptions included costs and probabilities associated with peanut allergic reactions.^4,5,13,20,21,22,23,24^ Model inputs are shown in Table 1. In these simulations, all children who received epinephrine or experienced anaphylaxis as a result of an allergic reaction to peanut were evaluated and treated in an emergency department as per currently recommended practices. All-cause age-adjusted mortality was applied to each cohort using 2013 US Life Tables.^22^ Food allergy fatality rates were based on a systematic review and meta-analysis published by Umasunthar and colleagues^5^ in 2013 that included 13 studies and 240 food allergy fatalities. The annual food allergy fatality rate in the pediatric population with food allergies (aged 0-19 years) was 3.25 (95% CI, 1.73-6.10) per million persons, with a fatality rate of 1.81 (95% CI, 0.94-3.45) per million persons reported in adults. Food allergy health state utility derived from standard gamble was included in the model,^19^ together with a 22% probability of peanut allergy resolution at 4 years of age, based on the natural history of peanut allergy reported from the population-based HealthNuts study^21^ birth cohort, in which 156 infants with challenge-confirmed peanut allergy underwent rechallenge (irrespective of risk profile) at 4 years of age. An accidental peanut exposure incidence resulting in symptoms was set at 7% per year with a severe reaction rate prompting emergency evaluation in 1% of patients each year, based on a retrospective review^20^ of 782 patients from 2 tertiary care allergy clinics. Models incorporated data from Robinson and colleagues,^4^ who described characteristics of patients presenting with anaphylaxis to the emergency department or urgent care center, noting only about two-thirds of patients with prior anaphylaxis had a prescribed epinephrine autoinjector device available at the time of their repeated anaphylactic event and describing a 35% rate of subsequent hospital admission for those treated emergently for anaphylaxis. No significant difference in admission rates was noted between patients who administered epinephrine prior to emergency department or urgent care center arrival. Costs of personal epinephrine autoinjector devices demonstrate variation, but as a base-case estimate, the 2016 average EpiPen (0.3 mg) pharmacy cost from a New England regional survey was incorporated ($715 [95% CI, $685-$743]) together with costs of emergency department visits and hospitalizations published by Patel et al^24^ and Gupta et al^25^ in 2011, expressed in 2018 US dollars. Additional costs of living with peanut allergy, including routine physician visits, visits with nutritionists and alternative health professionals, and grocery costs, were not modeled as they were standardized between cohorts and invariant regardless of presence or absence of a personal epinephrine prescription. Half-cycle correction was applied equally to each model with a cycle length of 1 year over the planned time horizon. Costs and quality-adjusted life-years (QALYs) were discounted equally at 3% per annum.^23^ This economic analysis conformed to the Consolidated Health Economic Evaluation Reporting Standards (CHEERS) reporting guideline.^26^ Statistical analysis was performed with TreeAge Pro and Microsoft Excel using descriptive statistics of distributed uncertainty expressed as 95% confidence intervals.

**Table 1.** Simulation Model Inputs

Variable	Model Reference (Sensitivity Range)	Source
US Life Tables	National Vital Statistics Reports, April 2017	Arias et al,^22^ 2013
Food allergy fatality	5-19 y: 3.25 (95% CI, 1.73-6.10; sensitivity, 3.25-33.00) per million person-years; ≥20 y: 1.81 (95% CI, 0.94-3.45; sensitivity, 1.81-18.10) per million person-years	Umasunthar et al,^5^ 2013
Rate of accidental peanut exposure and symptoms in individuals allergic to peanuts	7% per year (sensitivity, 5%-45%)	Neuman-Sunshine et al,^20^ 2012
Rate of ED visits for individuals allergic to peanuts	1% per year (sensitivity, 0.5%-3.5%)	Neuman-Sunshine et al,^20^ 2012
Personal epinephrine autoinjector carried, available, and administered at the time of anaphylaxis before arrival to ED	67% (sensitivity, 40%-100%)	Robinson et al,^4^ 2017
Hospitalization following ED visit for anaphylaxis	35% (sensitivity, 5%-45%)	Robinson et al,^4^ 2017
Spontaneous peanut tolerance	22% at age 4 y (sensitivity, 15%-30% at 4-22 y)	Peters et al,^21^ 2015
Personal epinephrine autoinjector, 2018 dollars	$715 (95% CI, $685-$743)	Shaker et al,^13^ 2017; US Department of Labor^23^
Hospitalization, 2018 dollars	$5899 (95% CI, $5732-$6066)	Patel et al,^24^ 2011; US Department of Labor^23^
ED visit, 2018 dollars	$691 (95% CI, $689-$693)	Patel et al,^24^ 2011; US Department of Labor^23^
Fatality risk increase without personal epinephrine available	10× (10× to 100×)	NA
Start age	Birth (birth to 5 y)	NA
Cycle length	1 y	NA
Annual discount rate	0.03 (0-0.03)	NA
Negative health state influence for food allergy	−0.09 (−0.02 to −0.11)	Carroll et al,^19^ 2009

Abbreviations: ED, emergency department; NA, not applicable.

The simulations compared patients who received annual individual prescriptions for self-injectable epinephrine with those who did not. Autoinjectors are only sold in the United States as a twin pack. Children younger than 19 years received 2 personal epinephrine twin packs (1 for home and 1 for school), with those 19 years and older receiving a single annual twin pack supply. In the base case, it was assumed that personal epinephrine was available and used by 67% of those individuals for who it was prescribed based on data reported by Robinson and colleagues.^4^ Adverse events from incorrect use of epinephrine were not included in the model. Outcomes measured were costs, QALY, and risk-specific fatalities. As the risk reduction associated with a personal epinephrine autoinjector prescription is unknown and not (to our knowledge) previously studied, we assumed a fatality risk reduction of 10-fold with a priori sensitivity analysis planned to 100-fold fatality risk reduction.^27,28^ Probabilistic analysis was performed with additional deterministic sensitivity analyses used to evaluate all variables. This study did not require institutional review board approval according to the policy of Dartmouth’s institutional review board.

## Results

**Figure 1.**
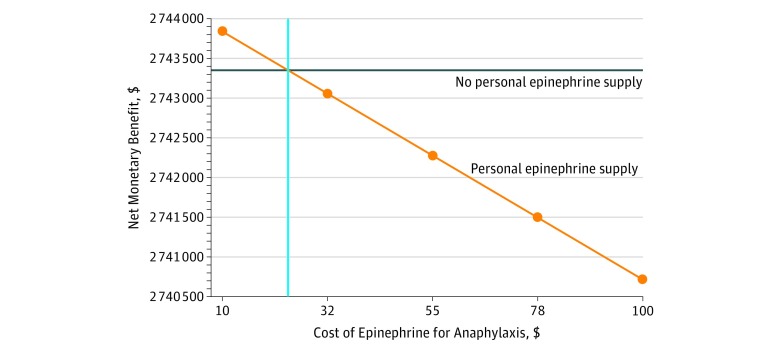
Sensitivity Analysis of Personal Epinephrine Cost Cost-effectiveness at a willingness-to-pay threshold of $100 000 per quality-adjusted life-year demonstrates a ceiling value-based cost of $24 for an annual personal epinephrine prescription in the base-case analysis.

The cost of anaphylaxis preparedness and treatment was higher in patients receiving annual prescriptions for personal epinephrine autoinjector twin packs ($25 478 [95% CI, $25 399-$25 557]) compared with those who did not receive annual prescriptions ($654 [95% CI, $645-$663]) (Table 2). Assuming a 10-fold risk increase from not maintaining a personal epinephrine autoinjector led to a small difference in the mean number of QALYs per patient (27.4446 vs 27.4335). The average rate of food allergy fatality was 0.00056 (95% CI, 0.000414-0.000706) per patient prescribed self-injectable epinephrine and 0.00148 (95% CI, 0.001242-0.001718) in those not prescribed a personal autoinjector twin pack. At a cost-effectiveness ceiling of $100 000 per QALY, an annual prescription personal epinephrine autoinjector priced at $715 was not cost-effective (incremental cost-effectiveness ratio [ICER] of $2 742 697 per QALY). However, at a cost of $24 per personal autoinjector epinephrine twin pack device prescription, the ICER for an annual prescription was $99 796 per QALY (Figure 1). If a 100-fold increase in food allergy fatality risk from failure to maintain an epinephrine autoinjector was assumed, value-based epinephrine cost could reach $264. If a 100% carriage and appropriate use compliance rate when treatment was indicated was assumed, the cost ceiling of personal epinephrine was slightly higher ($36 at 10-fold fatality risk and $393 at 100-fold fatality risk).

**Table 2.** Cost-effectiveness of Personal Self-injectable Epinephrine

Anaphylaxis Preparedness Strategy	$715 per Year				$30 per Year	
	Cost, $	QALY, No.	Risk-Specific Fatalities, No.	ICER, $	Cost, $	ICER, $
Annual personal epinephrine prescriptions (95% CI)	25 478 (25 399-25 557)	27.4446 (27.4247-27.4645)	0.00056 (0.000414-0.000706)	2 742 697	1685 (1675-1695)	161 810
No annual personal epinephrine prescriptions (95% CI)	654 (645-663)	27.4335 (27.4134-27.4536)	0.00148 (0.001242-0.001718)		NA	

Abbreviations: ICER, incremental cost-effectiveness ratio; NA, not applicable; QALY, quality-adjusted life-year.

**Figure 2.**
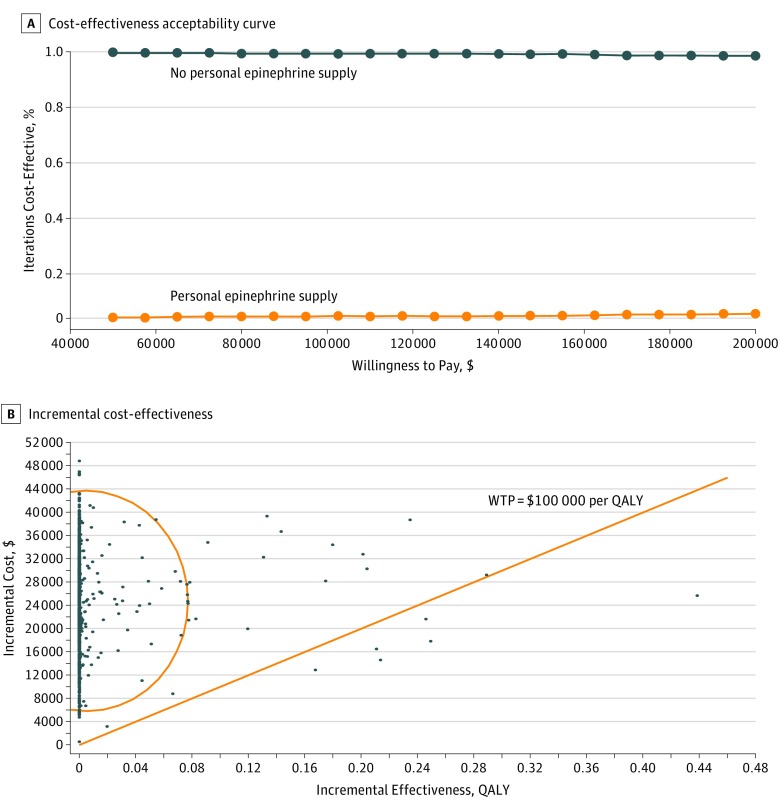
Cost-effectiveness of Personal Epinephrine Supply vs No Personal Epinephrine Supply A, Probabilistic sensitivity analyses performed on 1000 iterations of Markov simulations demonstrate personal epinephrine priced at $715 (95% CI, $685-$743) is not cost-effective across willingness-to-pay (WTP) thresholds of $50 000 to $200 000. B, Using probabilistic sensitivity analysis, more than 95% of 1000 iterations of Markov simulations (100 000 patients per group) were not cost-effective when annual personal epinephrine was priced at $715 (95% CI, $685-$743). QALY indicates quality-adjusted life-year.

**Figure 3.**
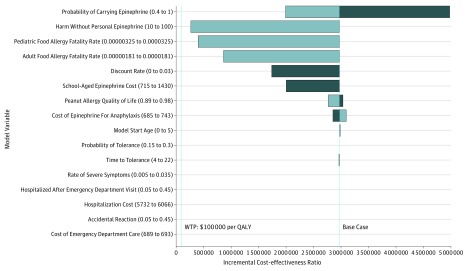
Tornado Diagram of Deterministic Sensitivity Analyses Dark bars denote values below the base-case assumptions and light bars denote values above the base-case assumptions. Values that deviate leftward of the base case do not cross the willingness-to-pay (WTP) threshold shown at $100 000 per quality-adjusted life-year (QALY). Values in parentheses on the y-axis represent the range of values simulated.

Probabilistic sensitivity analysis (n = 1000 trials) was performed with 99.4% demonstrating personal epinephrine priced at $715 (95% CI, $685-$743) was not cost-effective (Figure 2). At this price, personal epinephrine was not cost-effective across additional deterministic sensitivity analyses performed on all model variables (Figure 3). If a 10-fold increase in base-case food allergy fatality rates was modeled (ages 5-20 years, 32.5 deaths per million person-years; 21 years and older, 18.1 deaths per million person-years) and 100% carriage and appropriate use compliance rate was assumed when treatment was indicated, the ICER of personal epinephrine costing $715 was $199 958 and still exceeded value-based cost estimates. Personal epinephrine priced at $715 was only cost-effective ($28 190 per QALY) when a 100-fold fatality risk reduction was modeled together with 10-fold inflation of annual base fatality rates (32.5 and 18.1 deaths per million in children and adults). Even though evidence suggests personal epinephrine availability is unlikely to decrease hospitalization rates, we also evaluated cost-effectiveness if in addition to a 10-fold fatality risk increase, the probability of hospitalization was doubled for those who experienced a reaction and were without a personal epinephrine device. When both increased hospitalization and fatality risks were considered, the value-based price for personal epinephrine ceiling was $33 ($50 if it was also assumed all patients universally carry and use devices appropriately). Additional modeling that accounted for fatalities occurring before emergency department arrival did not significantly affect the cost ceiling estimate.

## Discussion

Epinephrine use for food allergy reactions is an important resource that is credited with saving lives, in particular in severe reactions such as anaphylaxis, where other medications will not address the underlying symptoms.^2^ Prescription of 2 epinephrine autoinjector twin packs (4 total devices) is a standard practice for school-aged food-allergic individuals.^2,29^ As awareness of anaphylaxis has risen in recent years, so has the cost of the autoinjector devices. The epinephrine cost controversy has been a well-publicized issue in the food allergy community and mainstream media, and it continues to seem enigmatic.^11^ At the crux of the matter is what is alleged to be a very high price for a device that uses technology developed in the 1970s and was subsidized by the US government, delivers a very cheap drug that is a century old,^17^ and was available for $100 or less prior to 2010.^8,9,30^ Currently, 3 device types are available, sold as 4 brands, representing 2 basic technologies. However, all deliver the same drug for the same indication.

Value-based pricing for epinephrine autoinjectors raises an interesting concept of having the outcomes of the device use justify the device price. Our analysis shows that these devices are significantly overpriced at current costs given outcomes based on fatalities prevented, even at exaggerated risk differentials for fatality and hospitalization or if current fatality estimates were increased logarithmically over their present rates. It should be stated that the base-case scenario presumed in the analysis of a 10-fold increased risk associated with not having an autoinjector is likely a very buffered estimate, and there are no data to substantiate that the risk is this high. In further sensitivity analyses, even at 100-fold increased risk and assuming a perfect carriage and usage rate, the price would still have to be less than $400 to be cost-effective. To our knowledge, no willingness-to-pay estimate for emergency treatment of a food allergic reaction has been calculated previously.

Fatality risk in food allergy is very low,^31^ and while epinephrine use is recommended for severe reactions, few recommended practices surrounding epinephrine use, such as preemptive injection prior to symptom development, default necessity to take any child receiving epinephrine to an emergency department immediately after injection irrespective of symptoms,^27,28^ or even the current practice of schools maintaining stock undesignated epinephrine at market costs, have been shown to be cost-effective (M.S. and M.G., unpublished data, 2018). There has been a sharp rise in anaphylaxis awareness and a recommendation for not only maintaining 2 units of epinephrine at all times, but having at least 2 twin packs as well.^2,29^ This resulting demand for devices has exacerbated availability shortages and likely creates an annual excess of unused devices. As anaphylaxis awareness has increased, the price of the devices has risen sharply in the past 6 to 7 years without much explanation.^8,13,30,32^ This culminated in a pricing crisis in 2016, when AUVI-Q was voluntarily taken off the market and EpiPen prices surged to their current levels of more than $600 per twin pack in the absence of its closest competitor.^8,9,13,30,32^ Interestingly, at the same time, the price of the Adrenaclick device (approximately $100) remained constant or even decreased slightly, although this device has never had strong market share.^33^

Epinephrine is the unequivocal drug of choice for anaphylaxis and other severe allergic reactions.^1^ However, the drug has to be affordable so that patients are neither priced out of epinephrine access nor forced to make other compromises to obtain an autoinjecting form of a drug that costs approximately $1.^17,32^ There are a few options to help prevent this. The first, obviously, is to lower prices. Our value-based model provides recommendations for such ranges. The second would be to examine the stability of the drug and the need to renew these devices every 12 to 18 months, which would shift cost from an annual expense to every 2 years and lower costs to some degree. At least 2 recent studies have suggested that epinephrine remains sterile and active after the listed expiration date on the device or package, and the FDA has temporarily extended the expiration to 24 months for selected autoinjector batches.^14,34,35^ A third option would be to refine the school-based public utility model for stock epinephrine and reduce the number of potentially superfluous devices sent to schools to supplement the stock model (provided the school has opted to stock an unassigned unit, if state law does not mandate a unit be maintained). We recently showed that in the Chicago Public School system this shifting alone could result in significant savings (M.S. and M.G., unpublished data, 2018). Fourth, having more device options on the market, providing more competition, could also help drive down device prices.

### Limitations

This study has limitations. Foremost, this is a simulation based on models and assumptions. As we have stated in previous analyses using similar assumptions of risk related to the difference between having and not having an autoinjector, we presumed a conservative estimate of a 10-fold risk, but there are no data to substantiate this as accurate because this particular risk reduction has not been studied and is unlikely to be studied owing to ethical concerns. In addition, there are very limited data regarding food allergy fatality; however, we used the best previously published estimates available and modeled higher-than-published rates in sensitivity analyses. Costs of adverse events from personal epinephrine use were not modeled, but this exclusion would only lower the cost ceiling of epinephrine. Patients experiencing a food allergy fatality assumed costs of emergency department care and hospitalization equally in each group, such that prehospital fatalities would not have introduced bias into either anaphylaxis strategy compared. Epinephrine device costs are not well documented in the literature (shifting rapidly per year and varying per pharmacy and insurance plan), and we used the best-available published data. Also, epinephrine prices may fluctuate over time, which could affect these estimates. However, to limit the issue of fluctuating price, we approached the question from the standpoint of determining a maximum price that reflected cost-effectiveness. Although value-based pricing was primarily based on fatality risk reduction, additional analysis exploring the effect of increased hospitalization risk continued to demonstrate a relatively low value-based epinephrine pricing ceiling ($33-$50 per year).

## Conclusions

Value-based pricing for drugs can allow pricing to be commensurate with proven outcomes.^18^ We explore a value-based pricing model for epinephrine and demonstrate that current autoinjector prices are not cost-effective in preventing an outcome of fatality from food allergy. However, at a price of $24 per year, personal epinephrine prescriptions become cost-effective. Access and fair pricing of potentially life-saving medication has been a continuing concern as technological breakthroughs deliver new innovations to autoinjector design. Incorporating value-based pricing into epinephrine market price decisions has the ability to add reasonable and rational benchmarks to a controversy that continues to baffle patients, practitioners, and payers.

## Notes

Supplement. **eFigure.** Decision Model
