# Supplementary material for: Association of Fatality Risk With Value-Based Drug Pricing of Epinephrine Autoinjectors for Children With Peanut Allergy: A Cost-effectiveness Analysis
Source: JAMA Netw Open. 2018 Nov 16;1(7):e184728. doi: 10.1001/jamanetworkopen.2018.4728 (PMC6324395; doi:10.1001/jamanetworkopen.2018.4728)
Supplement: Supplement. — eFigure. Decision Model [file jamanetwopen-1-e184728-s001.pdf]

Supplementary Online Content

Shaker M, Greenhawt M. Association of fatality risk with value-based drug pricing of epinephrine autoinjectors for children with peanut allergy: a cost-effectiveness analysis. *JAMA Netw Open*. 2018;1(7):e184728. doi:10.1001/jamanetworkopen.2018.4728

eFigure. Decision Model

This supplementary material has been provided by the authors to give readers additional information about their work.

© 2018 Shaker M et al. *JAMA Network Open.*

eFigure. Decision Model. Children receiving personal epinephrine autoinjector prescriptions were compared with those who did not receive annual prescriptions. Microsimulations of 100,000 subjects were evaluated in each strategy with costs, QALYs, and food-specific fatalities recorded.

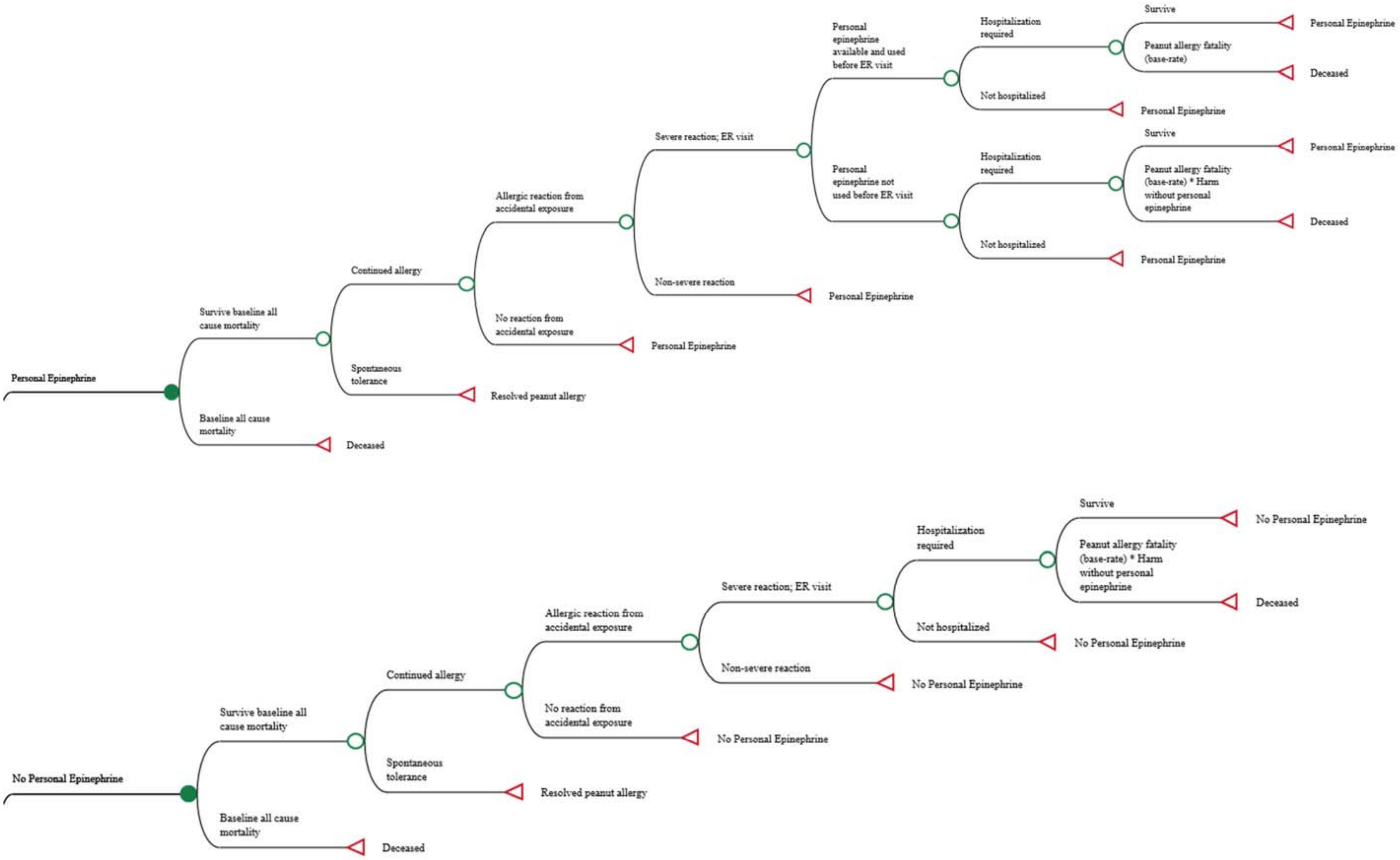

© 2018 Shaker M et al. *JAMA Network Open.*
